# Perceptions and experiences of frontline health managers and providers on accountability in a South African health district

**DOI:** 10.1186/s12939-020-01229-w

**Published:** 2020-07-01

**Authors:** Fidele Kanyimbu Mukinda, Sara Van Belle, Helen Schneider

**Affiliations:** 1 School of Public Health, University of the Western Cape, Robert Sobukwe Road, Bellville, Private Bag X17, Cape Town, 7535 South Africa; 2 Institute of Tropical Medicine, Nationalestraat 155, 2000 Antwerp, Belgium; 3 South African Medical Research Council (MRC)/Health Services and Systems Unit, Cape Town, South Africa

**Keywords:** Accountability, Equity, Frontline health workers, Maternal, newborn and child health, Qualitative research

## Abstract

**Objective:**

Public primary health care and district health systems play important roles in expanding healthcare access and promoting equity. This study explored and described accountability for this mandate as perceived and experienced by frontline health managers and providers involved in delivering maternal, newborn and child health (MNCH) services in a rural South African health district.

**Methods:**

This was a qualitative study involving in-depth interviews with a purposive sample of 58 frontline public sector health managers and providers in the district office and two sub-districts, examining the meanings of accountability and related lived experiences. A thematic analysis approach grounded in descriptive phenomenology was used to identify the main themes and organise the findings.

**Results:**

Accountability was described by respondents as both an organisational mechanism of answerability and responsibility and an intrinsic professional virtue. Accountability relationships were understood to be multidirectional - upwards and downwards in hierarchies, outwards to patients and communities, and inwards to the ‘self’. The practice of accountability was seen as constrained by organisational environments where impunity and unfair punishment existed alongside each other, where political connections limited the ability to sanction and by climates of fear and blame. Accountability was seen as enabled by open management styles, teamwork, good relationships between primary health care, hospital services and communities, investment in knowledge and skills development and responsive support systems. The interplay of these constraints and enablers varied across the facilities and sub-districts studied.

**Conclusions:**

Providers and managers have well-established ideas about, and a language of, accountability. The lived reality of accountability by frontline managers and providers varies and is shaped by micro-configurations of enablers and constraints in local accountability ecosystems. A ‘just culture’, teamwork and collaboration between primary health care and hospitals and community participation were seen as promoting accountability, enabling collective responsibility, a culture of learning rather than blame, and ultimately, access to and quality of care.

## Background

Accountability in health systems is perceived as key to improving health outcomes in low and middle-income countries (LMICs) [1, 2]. This was highlighted in the Millennium Development Goals (MDGs) and reiterated in the Sustainable Development Goals (SDGs), which not only advocate for more accountability to targets but also greater social accountability to communities and the public [3–5]. Increasingly, performance assessment systems and quality improvement initiatives are being implemented to improve the quality and efficiency of frontline health services provision through pathways of individual and collective accountability [6].

Accountability is an essential and intrinsic component of health system governance arrangements, concerned with the management of relationships between various actors [7]. In these relationships, meaningful accountability processes should address the systemic and structural drivers of inequity in health systems [8] in order to achieve universal health coverage (UHC). In this regard, UHC can only be achieved by fairness, accountability and transparency in the distribution of resources; by ensuring quality and access to healthcare, especially to marginalised communities; and by participation and building trust between health systems and the community. Through this pathway, accountability, understood as a driver and a ‘galvanizing force’, can thus improve health equity [8, 9].

Although accountability is emerging as a concept globally and nationally, its meaning is still unclear and complex [10, 11]. A review by Schillemans [10] describes the landscape of accountability definitions as a ‘true tower of Babel’, that is, confusing with contrasting meanings. There is consensus, however, on accountability as a set of institutional arrangements, organisational behaviours and accountability relationships [12]. Firstly, accountability is about the obligation to inform and explain actions or decisions taken to others, referred to ‘answerability’ [13]. Secondly, accountability is a relational concept, linking those who perform tasks (actors, agents) to those for whom the tasks are performed or who are affected by the tasks performed (principals). Accountability thus implies structures and processes (mechanisms) that mediate relationships and which are shaped by power [14]. These accountability relationships can be vertical within health system hierarchies [15], public/social mechanisms involving communities [16, 17] or horizontal accountability mechanisms between units or peers within the same system [18, 19].

Thirdly, processes of accountability can take many forms. Some focus on reporting requirements (answerability), others on sanctions, some on results or outcomes, others on organizational behaviour and processes [10]. A common approach to accountability is to assess how actors, programmes or policy are performing against agreed-upon targets or standards [20].

Writing from the perspective of maternal and newborn health, Hilber et al. [1] suggested the following broad definition, namely, that ‘accountability exists when an individual or body, and the performance of tasks or functions by that individual or body, are subject to another’s oversight, direction or request that they provide information or justification for their actions’.

In addition to accountability as an organisational mechanism as outlined above, Bovens [21] proposed viewing accountability as a ‘virtue’: a set of normative standards grounded in professional, ethical values for assessing the behaviour of public actors. As a virtue, accountability is positioned as a legal, ethical, and moral human attitude of obligation to communities that enables public trust and confidence [22]. It is associated with responsiveness and responsibility towards others, and a disposition towards transparency, fairness, and equity in actions and decisions [11].

Despite its importance and a growing number of accountability mechanisms in health services [23], very little empirical evidence exists on how frontline health managers and providers understand and experience accountability. Yet, ‘*increasing accountability of governments at national and facility level to ensure improvements in the quality of care by providers and managers depends not only on how mechanisms are enforced but also on how providers and managers understand accountability’* [1]. The available evidence suggests that notions of accountability will vary by profession (doctors versus nurses; clinicians versus support staff), within hierarchies (managers versus providers), and between health system and community actors. This variation also relates to the competing values and multiple internal and external loyalties typical of a service delivery environment [24].

As part of a PhD study exploring the forms and functioning of accountability mechanisms for maternal, newborn and child health (MNCH), this paper explores the perceptions and daily working experiences of frontline public sector managers and providers regarding accountability in a South African health district, serving a rural community with a higher level of poverty relative to the rest of the country. Perceptions relate to the mindsets and understandings, while experiences relate to practices (by the providers themselves or others). In contrast to the abstract formulations of accountability in the literature and in global and local policy, the paper examines the everyday, ‘real world’ understandings of accountability of health providers and managers at the receiving end of accountability strategies and how they relate to it as a practice to ensure the quality and performance of primary health care (PHC) and district health system (DHS), key to strengthening equity.

The paper focuses on maternal, newborn and child health (MNCH), as a programmatic area where accountability mechanisms were established in South Africa’s health services over a number of years, especially in the period of the MDGs [25]. More recently, a dramatic rise in medical litigation linked to maternal and neonatal services has been attributed to the lack of accountability in environments which do not ensure the delivery of safe, respectful and effective health care [26].

## Methods

### Study design

We conducted a qualitative study of how frontline health managers and providers perceive and experience accountability using maternal, newborn and child health as a tracer. Our methodology followed a phenomenological approach that seeks to examine and represent the meaning systems and lived experiences as expressed by the respondents [27]. This implied that the research aimed to get into ‘their worlds’ to gain knowledge and new insights and to stay true to the words and forms of representation of the respondents themselves [28, 29]. This requires the researcher to be ‘*observant, attentive and sensitive to the expression of experiences*’ and questioning their understanding of respondents’ narratives [30].

### Setting

South Africa is a middle-income country with a quasi-federal political system consisting of the national sphere, nine provincial governments and 52 health districts. South Africa has been regarded as a poor performer with respect to maternal and child health outcomes, and a number of accountability strategies were implemented in the health system to address this. They include the Confidential Enquiry into Maternal Deaths (CEMD), the Perinatal and Child under-five Problem Identification Programmes (PPIP and CHIP), and a range of other clinical governance and quality assurance measures. This study was conducted in Gert Sibande District, one of three districts of Mpumalanga Province, situated in the north-east of South Africa. The District has a population of about 1.1 million, with the majority (61%) living in rural areas (Massyn et al., 2017). The District comprises eight district hospitals, one regional hospital and 76 primary health care (PHC) facilities, distributed among seven sub-districts. These public health facilities serve poor rural communities, including migrants and farmworkers, and are for the most part free at the point of use.

### Sample and data collection

We used a purposive sampling method to select key informants from two of the seven sub-districts and the district office. The sub-districts were selected in a prior study as representing the range of buy-in to one particular MNCH accountability strategy [31]. Informants were sampled among frontline managers and providers involved directly in MNCH, and among those indirectly associated with accountability for MNCH, using a snowballing approach. They consisted of the following: district programme managers and members of the district maternal and child health clinical specialist team (DCST), hospital CEOs, operational (unit) managers from PHC facilities and district hospitals, professional nurses, allied health professionals, emergency service personnel, community representatives (chairpersons of hospital boards), and trade union representatives (total 58 respondents).

Strategies for data collection were discussed and agreed by all authors. Data were collected using semi-structured in-depth, individual interviews and one focus group discussion with PHC operational managers. In addition to a few demographic details, interviewees were asked the following four open-ended, exploratory questions: What does accountability mean for you and how do you experience it in your daily practice? To whom are you accountable and for what? What are the barriers, facilitators and challenges to current accountability for MNCH in practice? What can be done to improve accountability?

Interviews were conducted by the first author as part of a wider study, which also involved repeated visits, immersion and observations of accountability processes over 16 months. The average time of each interview was 45 min (ranging from 22 to 89 min). The interviews and focus group discussion were audiotaped and, with respondents’ permission, transcribed verbatim. During and after the interview the interviewer took notes and summarised the interview on a coversheet designed for that purpose. All audio files and transcripts were reviewed by the authors to ensure quality.

### Data analysis

Data from the open-ended questions were organised, coded and analysed inductively using Atlas.ti (Version 8), and a thematic approach was used to analyse the data. In the first step, each respondent’s transcript was read several times together with listening to the recording to form an initial understanding of the expressed sense of accountability. Codes were developed iteratively based on the content of the interview guide and emerging insights. An initial code list was identified by all authors and tested on selected transcripts from the three research sites. After discussion, consensus and validation of the code list, the remaining transcript coding was done by the first author. Next, all transcripts were coded and significant statements (quotations) representative of the perspective or experience extracted. Codes with similar patterns were grouped into themes and similar themes were organized into categories. Finally, the findings were integrated into a comprehensive description of the concept of accountability that was presented to respondents in various meeting platforms for them to verify and validate the results.

#### Validity, truthfulness and ethical considerations

The researchers sought to apply the ‘bracketing’ principle of phenomenology by deliberately putting aside their pre-existing knowledge and adopting a ‘not-knowing’ attitude ‘to maintain the curiosity in the participants’ [32].

The periods of immersion and observation, which formed part of the wider study, not only built trust with participants but also enabled the authors to contextualise and interpret the material from the interviews. Apart from the regular feedback and discussion of the findings during follow-up meetings in the district, iterative processes between the first author (PhD student) and his co-authors (PhD supervisors) through ongoing communication and continuous questioning of the understanding of data and reviewing of findings, provided opportunities for minimising descriptive and interpretive biases.

## Results

Table 1 summarizes the characteristics of key informants from the two sub-districts and the district office. Of a total of 58 participants, 36 (62%) were female, 43 (74%) were managers (senior and mid), and 3 (5%) were chairpersons of the hospital boards representing the community. Thirty (51.7%) respondents were nurses and 9 (15.5%) doctors; their experiences vary from less than 1 year to over 10 years at the time of this study.

**Table 1:** Characteristics of key informants

	n (%)
**Sex (*n*= 59)**	
Female	37 (62.7)
Male	22 (37.3)
**Category (*n*** **= 59)**	
Doctors	10 (16.9)
Nurses	30 (50.8)
EMS	1 (1.7)
Allied, Dieticians, Social workers	7 (11.9)
Community representative	3 (5.1)
Information Officers	3 (5.1)
Pharmacist	1 (1.7)
Corporate (HRM, Asset, Laundry)	4 (6.8)
**Function category (*n*** **= 59)**	
Manager	44 (74.6)
Non-manager	12 (20.3)
Community representative	3 (5.1)
**Duration in position (*N*** **= 44)**	
Less than 1 Year	3 (6.8)
1–3 years	8 (18.2)
4–7 years	17 (38.6)
8–10 years	5 (11.4)
Over 10 years	11 (25.0)
**Level of care (*n*** **= 59)**	
District Office	13 (22.0)
District Hospital	33 (55.9)
Sub-District Office	2 (3.4)
Ideal Clinic	11 (18.6)
**Interview types (*n*** **= 59)**	
Individual	50
1 FGD of 9	9

In the following sections, we provide a detailed description of what the respondents understood or perceived as accountability, what they experienced as the barriers and enablers of accountability, and their recommendations for improving accountability. To maintain the credibility of our findings, identified themes are presented with a short descriptive text and illustrated with representative quotes [30].

### Defining accountability

Frontline health managers and providers in the district had well-formulated views and definitions of accountability, following Hilber’s [1] key attributes with words such as being ‘responsible’, ‘answerable’ and ‘transparent’ frequently invoked (Table 2). Formal, bureaucratic versions of accountability existed alongside ideas of accountability as a professional virtue and a product of intrinsic motivation (referred to by one respondent as ‘passion’), as proposed by Bovens [21].

- *Accountability as being responsible*

**Table 2:** Frontline managers’ and providers’ definitions of accountability

Definition	n (%)
Responsibility	32 (39.0)
Answerability	19 (23.2)
Compliance (Norms, Guidelines, Targets)	9 (11.0)
Transparency/Reporting	7 (8.5)
Realise promise/Provision of Quality Care	5 (6.1)
Sanctions	4 (4.9)
Performance	3 (3.7)
Obligation to Update Knowledge	1 (1.2)
Provision of Strategic Leadership	1 (1.2)
Recognise hierarchy	1 (1.2)
**Total**	**82 (100)**

(Note: *n* = Number of times the term was mentioned from a total of 82)

Accountability was most often referred to as being responsible for any decision taken, and ‘act or omission’ in the line of duty. Being responsible took different forms, from general awareness and internal disposition to more specific notions linked to management in hierarchies.

A hospital Chief Executive Officer (CEO), as the main ‘accounting officer’ of the institution, indicated his awareness of his ultimate responsibility for all actions taken in the hospital.

*For all the good things I am accountable and even for all the bad things and also for omissions of which our officials might have been involved in … [Hospital CEO].*

Reflecting a similar understanding at an operational level, an information officer described accountability as taking responsibility for doing one’s work without mistakes.

*… Everything that you are doing you are ... we are responsible for it; you must make sure that there’s no mistake there … accountability means you must take full responsibility [Information Manager].*

A senior nursing manager, on the other hand, understood accountability as assigning responsibility to ‘subordinates’ in a management line, while retaining accountability.

*‘Accountability according to my understanding … is assigning responsibility to your immediate subordinates, but as the accounting person you don’t assign accountability, accountability remains with you’ [Nursing Manager].*

Accountability was also referred to as a process of assigning responsibility (fault) to system actors in cases of wrongdoing or negative outcomes.

‘*Whose fault is it that someone got malnourished or died or anything like that?’ [Dietician].*

Such wrongdoing could invite sanction:

*‘ … The Minister said where we are going there will be time if anything is really happening in the hospital, [an] investigation done [which] finds that there is something like negligence, so and so will be accountable; and when you are accountable, people they will even lose maybe a salary … ’ [Manager].*

b. *Accountability as being answerable*

Accountability was also perceived, alongside responsibility, as being able to answer or explain, referring to the obligation to justify any decision or action taken that resulted in the observed outcome for the patient or the system. As with responsibility, the notion of answerability was described both as a personal attribute and as compliance to external rules, as implied by the two contrasting accounts below:

*‘To be accountable is to be able to answer, to be answerable, to be able to answer for the actions that you have taken, to be able to give the reasons why you did what you did and the way you did it. So that to me that is accountability’ [Operational Manager].*

*‘Accountability means that you agree to abide by the protocols, the prescripts, the guidelines and whatever that you do, it is [judged] against what the protocols or guidelines are saying … ’ [Manager].*

c. *Accountability as a virtue*

Underpinning ideas of responsibility and answerability as a personal attribute, the narratives of respondents made frequent reference to accountability as driven by personal values, intrinsic motivation and professional commitment.

‘*To me, it’s a sense of duty, accountability means a sense of duty, sense of urgency, effectiveness, sense of accountability itself and sense of responsibility as well. To me, all that forms part of accountability’ [District Programme Manager].*

As a moral value or virtue, accountability transcends professional knowledge and experience to embrace ‘knowledge with passion’, and collective commitment to the provision of quality care, as expressed in one sub-district.

*‘… one of the key things helping this sub-district is to have people with passion in those wards … like here in maternity ward Sister [name], paed’s ward Sister [name], the operational manager; to have people with a passion at the same time experience, because they’ve been here for a long time. They have the experience, they have knowledge. If you have the knowledge it’s good. But if you have knowledge and passion then you make a difference … ‘[Allied Health Manager].*

Accountability is being sensitive to patient needs, particularly to the patients served in public health facilities within the district.

*‘You need to stand up and go to the waiting area and check; that also makes people more comfortable. If they know that she [nurse] has seen me, she knows about me, every time she comes out, she says I have noticed you, see you now, now, it makes people comfortable, they can relax, they know they will be helped’ [PHC Manager].*

Finally, as a virtue, accountability is perceived as a response to trust that the community placed in the health system.

*‘I’m accountable to the patient that I’m giving the service to. Because I’m accountable to her, that I know that when she left her home to come here, she trusts us and she is putting all her trust to me, so I must do justice to her, I’m accountable to her’ [Operational Manager].*

### The multiple directions of accountability

When asked to whom they were accountable, respondents typically saw themselves as being accountable simultaneously to other health system actors, upwards and downwards in a hierarchy, horizontally to peers, and outwards to patients and communities. Their understandings thus encompassed notions of both internal and external accountability.

*‘Firstly, I’m accountable to the patient that I’m giving the service to. And also, I always tell myself I’m accountable to the colleagues that I’m supervising because whatever good and bad things that they are doing it will reflect back to me[ …*] *And all in all, I’m accountable to the Department because they put me here as they’ve trusted me that I’m going to represent them in a good way’ [Operational Manager].*

For some, accountability involved a reciprocal relationship of ‘giving hope’ and responsiveness to staff downwards in a hierarchy:

*‘Administration-wise … apart from accounting to the District Manager, the head of the department and the MEC for Health, at the end of the day I account to the community [ …*], *as well as the staff, meaning here I must give hope to the staff because you see, there are lots of challenges and internal issues that need to be attended to, your shortage of staff, your lack of equipment, your shortage of skills, your need for training...’ [Hospital CEO].*

Accountability was expressed as a relationship, both to immediate line managers and patients and a wider system and ‘citizens’.

*‘Workwise, I account to the District Manager in terms of meeting all the objectives that I have to meet according to the key performance … I am [also] accountable to the citizens of the country for one reason - they are the funders of the whole government project’ [District Programme Manager].*

Community representatives on Hospital Boards described a complex mix of accountability relationships involving communities, political principals (the Member of the Executive Council (MEC) - the Provincial Health Minister) and trade unions.

*‘My accountability, or our accountability, as board members I think, is in two ways. We account to the community, that’s a very critical role. And the second one, we also account to the MEC and you would understand that because the MEC is directly elected by the community’ [Hospital Board Chairperson].*

*‘ … the unions and also the community members, there is no way that you can disregard what they say’ [Hospital Board Chairperson].*

Finally, linked to the narratives of accountability as a professional virtue, frontline managers and providers often described a relationship of accountability to the self.

*‘First, I’m accountable to myself … because you know every time you save a life … I don’t say it’s happiness, it’s something like it’s a fulfilment, you go back home and you say I saved a life [ …*] *I think the first one is to myself, [then] to the community, to the management’ [Medical Officer].*

### Enablers and barriers of accountability

While having clear ideas about definitions and directions of personal accountability, interviewees saw the everyday practice of accountability as embedded in a wider set of organisational relationships and processes, where leadership styles, communication, team-work and community engagement were key factors.

- *Leadership and management styles and practices*

Respondents identified hands-on, accessible leadership styles as a key to accountability. One hospital CEO described his ‘open door policy’ as follows:

*‘ … having this open-door policy I speak even with the cleaner down there, I am not saying no, no I won’t speak to you I will only speak to your supervisor or whoever just to be in contact with everyone …*. *when you are in touch with your people you know they can come to you at any time, phone you, talk to them, go to where they work, look at the area where they are working, you will understand the situation’ [Hospital CEO].*

Variation in the involvement and closeness of the leadership to staff within the district was described by another frontline provider as follows:

*‘The leadership is very important. For example, in Hospital A, I worked also in that hospital, the leaders are there somewhere, and you, you are your side. It is very different from Hospital B, the leaders are very involved starting by the CEO, you could see that every time he’s got an occasion he attends the meetings; Dr [clinical manager], once I take the phone and say, ‘mommy I am in a difficult situation’ she will arrive. You see that the leadership is very involved’ [Medical Officer].*

Leadership styles and practices were most evident in the manner in which ‘adverse events’ such as maternal deaths were responded to at district and higher levels. While these events were infrequent, the attention brought to them, and the way responsibility was assigned and sanctions applied, was watched carefully by frontline actors, setting a wider tone for perceptions of accountability at sub-district and facility levels. Respondents described instances of both unfair, harsh punishment and impunity in response to adverse events.

*‘In this office yes... others were suspended for something that they did not do’ [District Programme Manager].*

*‘ … but when it comes to sanctions, why these ones are punished this way, I can say it’s a punishment, why those ones are not punished, you know this discrepancy … ’ [Medical Officer].*

Politically connected players could escape sanction:

*‘ … politics is mixed with the administration … so, that compromises accountability a lot; if people are doing wrong it’s difficult to reprimand them; because if you go to your external structure, that person is the secretary or the chairperson in your political branch’ [District Programme Manager].*

Practices of impunity created the conditions for malpractice suits, while unfair punishment engendered a climate of fear of reporting:

*‘ … When they are suing the hospital, they are not suing you as an individual. That’s where accountability is coming in because people are thinking that if something happens it’s fine the government will resolve it for me, and they can continue doing the very same things’ [Manager].*

*‘Most of the time, people, they think that maybe when you report, the punishment is coming … ’ [Manager].*

b. *Strengthening provider motivation and skills*

A ‘people-centred’ approach was seen as a key enabler of accountability by a senior clinician in the district.

*‘The things in health are run by people; a machine can help but it’s the people who are delivering the service … If we have the right people with the right training, the right updating [of knowledge] and everything, also with the right motivation that they are really attended to in proper way as human beings, then for me it’s almost impossible not to reach the point’ [DCST member].*

Provider motivation could be strengthened in several ways, including responsiveness to needs, acknowledgement of good performance and respectful interactions:

*‘Motivation is a very wide word. I don’t want to say we’ll give you more salary, we’ll give you a house. Motivation sometimes is to attend the people’s needs, to have the proper equipment, to work in proper conditions, and to tell them ‘thank you, you are doing well’ when you are doing well; And when they are not doing well to call their attention in a respectful way. Motivation is not necessarily about spending money or to give more [material] things; motivation for a human being can be simple’ [DCST member].*

Of these, acknowledgement of good performance and achievement was particularly valued.

*‘ … I spoke to him [HOD] and asked [ …*] *I would like you before you leave to go and say something nice to my nursing staff. He asked me why, I said you know since I’ve been here, we never had any maternal death, and those guys need at least to hear from you a ‘thank you’. He came and spent some minutes with them, he thanked them and it was very good’ [Medical Officer].*

Alongside strengthening their motivation, improving accountability required equipping providers with the right knowledge and skills.

*‘So, I think knowledge is power … If we are given money to improve accountability, I think step number one will be to give people the information, knowledge. Because once people have knowledge on that particular programme or on that particular work that they are doing, they will be able to account better and even the superiors or the accounting officer would be able to hold them accountable because they’d be having knowledge’ [Allied Health Manager].*

c. *Communication and teamwork*

Respondents identified effective communication and collaborative teamwork and support systems between levels of care as an important element in strengthening collective responsibility and a ‘no-blaming’ environment.

‘*Because previously we were having that thing that PHC would point at the hospital, we, when we have done wrong, we will point it back to the PHC, and we have been pointing it back because they are not in our meetings; now we are together’ [Operational Manager].*

*‘ … We need to have a support system; [ …*] *first we must have a good referral system in a way that when I have a problem I should have a backup. A good referral system includes first a very good team, a district hospital, very good communication, very good transport system. It’s a holistic system that involves everybody, involve the community’ [Medical Officer, SD2].*

Conversely, the lack of communication was experienced as a barrier to accountability that affected the quality of care and created a culture of blaming and shifting of responsibility, as these two quotes from one facility illustrate.

*‘I have to be honest … I identified that there is no link, there is no communication in terms of the hospital as well as the PHC’ [Hospital CEO].*

*‘There is a culture of blaming within the hospital that brings the feeling of embarrassment; there is also a behaviour of policing behind your back, like people watching you report on any mistake’ [Medical Officer].*

Finally, unity and teamwork among key managers in hospitals (the ‘Big Five’) were important in consolidating accountability within the organisation.

*‘I think the key people are the ‘Big Five’ at the hospital level; the CEO, the nursing service manager, the corporate manager, then finance and the clinical manager [ …*] *even though I’m a nursing service manager, but when I go to a unit, I will make a doctor account the same way the clinical manager will make a nurse account for his/her action. So probably the teamwork between the Big Five is important to ensure that people are accountable’ [District Programme Manager].*

d. *Engaging communities and trade unions*

Openness to communities and representative structures such as trade unions was a recurring theme as shaping the accountability ecosystem.

*‘We normally conduct community dialogues, where different stakeholders come together [ …*], *an example regarding the late booking of the antenatal care; people are voicing out what can be done and they are voicing out why people are not booking early for the antenatal care. Then after the dialogue, we sit down and plan for the activities that can improve the situation together with the community’ [District Programme Manager].*

Respondents expressed various views on trade unions as a ‘voice’ for accountability.

*‘ … organised labour formation, that for me is very key because it also contributes to the wellbeing of the entire operations within a hospital setup’ [Hospital Board Chairperson].*

On the other hand, trade unions were also described as powerful, but problematic players.

*‘No, their voices are not for pushing for improvement. Their voices are more for getting people angry; If they use that effort, you would see a different place, if they use that effort to try to improve and try to motivate and try to get people to do the right thing’ [PHC Manager].*

## Discussion

This paper provides a descriptive account of how public sector frontline health managers and providers perceive and experience accountability in the context of a district health system serving a poor rural community.

The study found that these health system actors had well-established ideas about, and a language of, accountability, in contrast to the ‘inability to define the concept of accountability’ reported in a study of health workers in another South African Province [33]. However, as described by Baumann et al. [34] in the Canadian setting, respondents did not present a single or common understanding of accountability. On the one hand, they described accountability as responsibility, answerability or compliance, showing the internalization of accountability as an ‘organizational mechanism’ involving answerability for ‘acts and omissions’ within hierarchies. On the other hand, they also saw accountability as a moral value and intrinsic professional attribute, described by Bovens et al. [11] as a virtue. These authors suggest that making a distinction between accountability as ‘mechanism’ and as ‘virtue’ is the first step in addressing the conceptual confusion in studying accountability.

Accountability as a virtue is a reflection of public-interest values; it is linked to ideas of healthcare as a profession, involving public proclamations (through oaths) of commitment and dedication, and the suppression of self-interest for the wellbeing of the peer human beings as recipients of healthcare [35]. Similarly, even though study participants were very aware of their place in hierarchies, the majority simultaneously expressed strong accountability to patients and communities, to peers and the ‘self’ as a professional. Their narratives reflected their collective positioning in a classic professional accountability model described by Emmanuel and Emmanuel [36].

This wider understanding of accountability is an asset for better understanding of health inequities and social determinants of health, and for promoting the acceptability and quality and ultimately, equity, of health services. This notion is important to recognise and nurture in strategies to strengthen accountability and improve the quality of healthcare at the frontline [1]. The findings also suggest that frontline providers and managers are less in need of further training on accountability, values clarification or new accountability mechanisms given the crowded nature of the accountability space [23]. However, interviewees were all in agreement that they needed enabling local environments that better support their practices of accountability [26].

The respondents in the district recognized the following as enablers of accountability, shaped by the local context of each sub-district and facility: collaborative, multidisciplinary teamwork; good relationships between levels of care, community participation; and an open leadership style. Alongside these elements was paying attention to provider motivation, including recognition for good performance and words of encouragement, respectful interactions, sound human resources practices, investment in skills development and support systems that are responsive to needs. Such reciprocal processes of accountability between management and spheres of practice, described by Elmore et al. [37], are key to performance.

Respondents also described several challenges to accountability, including blaming and shaming cultures, and instances of perceived unfair sanction for some actions while others continued with impunity. As observed by Aveling et al [38], sanctioning individuals when systems are inadequately designed or poorly functioning may be masking deeper ‘organisational pathologies’. Van Niekerk also alluded to healthcare workers being unfairly called to account daily on tasks that fell beyond their scope of practice [33]. Therefore, formal accountability procedures do not automatically lead to better health equity if socio-economic inequities and health system structural failures are not adequately addressed as root causes.

The respondents argued less for doing away with individual accountability so much as fair approaches to sanctioning, and more broadly, the development of environments that promote the ‘opportunity to be good’ [38]. Such an approach affirms ethical and moral responsibility for actions and behaviours of frontline health professionals while also creating conducive organisational environments and norms of fairness and collective responsibility in which individuals may be held accountable [38].

Interviewees readily provided examples and experiences where there had been a shift from a blame culture to one known in the health care safety literature as a ‘just culture’ [39]. Respondents were very aware of the elements of such a just culture, including the organisational and managerial practices which enabled accountability and strengthened performance, and how these were configured in the individual spaces of the district. This suggests considerable potential for improving accountability through lesson learning within the district. Moreover, an internal, just culture will promote equity in the provision of health care. However, local and provincial contexts where administrative and political decision-making processes are blurred, an excessive focus on compliance rather than relational approaches to accountability from higher levels of the system [23], and a growing fear of litigation may all constrain the expression of just cultures at a district and sub-district level.

Finally, any plan to improve accountability for better MNCH outcomes should include strengthening community participation which is recognized elsewhere as a key mechanism to increase provider accountability [40]. In this regard, the WHO’s Partnership for maternal, newborn and child health (PMNCH) recommends that effective accountability mechanisms should ensure transparency and inclusiveness

### Limitations

Accountability is a sensitive subject, and respondents’ accounts may not fully represent the reality of their practices. The idealistic statements of accountability and ethics from respondents could have been what they thought to be the right answer reflecting a social desirability bias in their responses. We sought to minimise this by prolonged immersion in the field and supplementing formal interviews with informal conversations and observations (reported more fully in [23]). Basing the research on respondents’ self-reports and accounts could have led to an overstatement of phenomena and introduced a common method bias [41]. Effort was made to include as many respondents as possible and to consistently probe answers throughout the semi-structured interviews. Possible interpretive bias was dealt with through the lead researcher’s reflexivity and questioning from the other authors. Furthermore, each author engaged separately with the data from own perspective and sought to identify key themes separately which were then discussed and agreed on collectively.

## Conclusion

Frontline providers and managers in this rural district of South Africa had well-established definitions of and views on how to strengthen accountability for performance. While not negating the role of individual accountability, they pointed to system-related factors driving inequity and the need for promotion of a ‘just culture’ [39] of accountability, learning and improvement at the individual and organizational level. This has important implications for promoting equity in access and ensure that the system is leaving no one behind.

**Table:** 

Significance and contributions	
**Problem in what is already known** Accountability is emerging as a key concept in health systems globally and nationally, and particularly in relation to Maternal, Neonatal and Child Health. How frontline providers perceive and experience the everyday practice of accountability is not well understood. **What this Paper Adds** ▪ Frontline providers have varied understandings of accountability, but express strong professional notions of responsibility, answerability and accountability as a ‘virtue’. Their everyday practice is deeply influenced by the organisational environment. ▪ Formal accountability procedures do not automatically lead to better health equity – On the contrary, it might lead to ‘naming and shaming’ among public workers, without adequately addressing structural determinants. ▪ District and primary health care systems play an important role in strengthening equity of access, availability and quality of healthcare services. Countries facing similar issues of disparities in access to quality health care need to revisit how frontline healthcare workers conceptualize formal and informal accountability as part of their job and professional identity. ▪ The micro-contexts of accountability are not uniform between various local settings, and this variation provides an opportunity to strengthen accountability and improve the quality of care provided through lesson learning.	

## Data Availability

Not applicable.

## Abbreviations

CEO: Chief Executive Officer
CEMD: Confidential Enquiry into Maternal Deaths
CHIP: Child under-five Problem Identification Programme
DCST: District Clinical Specialist Team
MNCH: Maternal, Newborn and Child Health
PPIP: Perinatal Problem Identification Programme
